# Development of reference assignment in children: a direct comparison to the performance of cognitive shift

**DOI:** 10.3389/fpsyg.2014.00523

**Published:** 2014-05-30

**Authors:** Taro Murakami, Kazuhide Hashiya

**Affiliations:** ^1^Department of Human-Environment Studies, Kyushu UniversityFukuoka, Japan; ^2^Faculty of Human-Environmental Studies, Kyushu University of FukuokaJapan

**Keywords:** preschooler, pragmatics, inference, reference assignment, executive function

## Abstract

The referent of a deictic embedded in a particular utterance or sentence is often ambiguous. Reference assignment is a pragmatic process that enables the disambiguation of such a referent. Previous studies have demonstrated that receivers use social-pragmatic information during referent assignment; however, it is still unclear which aspects of cognitive development affect the development of referential processing in children. The present study directly assessed the relationship between performance on a reference assignment task (Murakami and Hashiya, in preparation) and the dimensional change card sort task (DCCS) in 3- and 5-years-old children. The results indicated that the 3-years-old children who passed DCCS showed performance above chance level in the event which required an explicit (cognitive) shift, while the performance of the children who failed DCCS remained in the range of chance level; however, such a tendency was not observed in the 5-years-old, possibly due to a ceiling effect. The results indicated that, though the development of skills that mediate cognitive shifting might adequately explain the explicit shift of attention in conversation, the pragmatic processes underlying the implicit shift, which requires reference assignment, might follow a different developmental course.

## Introduction

The referent of a deictic embedded in an utterance or sentence is often ambiguous. We communicate with others by interpreting the intended referent embedded in an utterance. However, interpreting another's referential intention is hardly achieved by a simple decoding process (Sperber and Wilson, 1986). The receiver must identify the intended referent based on a preceding situation or context. Reference assignment is a pragmatic process that enables disambiguation of a referent.

Previous studies have demonstrated that by age 2, children begin to use various non-verbal cues to determine the referent, such as the focus of the other person's attention (Baldwin, 1991), previous interactions with the other (Moll and Tomasello, 2007; Moll et al., 2007), the other's expression of preference (Repacholi, 1998), or the other's expression of glee or disappointment (Tomasello and Burton, 1994). Other researches have further demonstrated that children of the same age interpret an ambiguous request for absent objects, such as “Can you give *it* for me?” (Ganea and Saylor, 2007) or “Where's the ball?” (Saylor and Ganea, 2007), by reflecting on previous interactions with the experimenter that concerned particular objects. These studies agree in the sense that 2-years-old children have acquired the ability to use the relevant non-verbal information that has been gained through previous triad communications (self-object-other) in the process of interpreting an ambiguous referent.

Clark and Marshall (1981) pointed out the importance of linguistic evidence in processes where the receiver uses some form of information in interpreting a referent. Linguistic evidence could be termed as what the two persons have jointly heard, said, or are now jointly hearing as participants in the same conversation (also see Clark et al., 1983). In particular, the receiver must use contextual information from a shared conversational background to interpret the anaphoric expressions. With regard to the development of this ability, Ganea and Saylor (2007) demonstrated that 15- and 18-month-olds used the speaker's previous reference to an absent object to interpret the request.

However, in verbal communication, contextual redundancy often results in ambiguous referent interpretation because an object inevitably contains multiple aspects of information (name of object, color, function, and so on). When the labeling situation becomes ambiguous and the child has to determine from three or more alternatives which object is being labeled, 2-years-old interpret the novel words based on prior shared experiences with the experimenter (Akhtar et al., 1996; Diesendruck et al., 2004; Grasmann et al., 2009). Our previous study also indicated that 3-years-old children do not always use linguistic information from prior conversations retrospectively as a cue to interpret an ambiguous “How about this?” utterance (Murakami and Hashiya, in preparation). In this “reference assignment” task, 3-years-old children did not (though 5-years-old children did) refer retrospectively to the preceding linguistic context to identify the referent of an ambiguous utterance in the situation where the aspect to be referred in conversation was systematically changed (from shape to color or vice versa). The 3-years-old children, relative to 5-years-old, were also less proficient at shifting the referential aspect explicitly.

To effectively disambiguate an ambiguous referent, the receiver must attend to the same aspect as the sender. Evidence suggests that the ability to attend based on a verbal instruction might depend on the ability to perform a cognitive shift (directing attention from one aspect to another) (Murakami and Hashiya, in preparation). If the ability to interpret the ambiguous referent is based on the ability to track the interactions with the other, one could predict that children who are better at shifting their focus of attention should assign the referent more effectively when reflection on prior interactions with the other is useful. Primarily because of the close correlation between performance on “mind-reading” tasks, like False Belief, and the DCCS, the common underlying mechanism in terms of executive function (EF) is regarded as “domain-general” ability. To further examine this “domain-general” hypothesis, it should be determined whether EF predicts referent disambiguation performance. However, the relationship between these abilities has not yet been examined. Therefore, the present study directly assessed the association between reference assignment task and dimensional change card sort (DCCS) task performance in 3- and 5-years-old children.

The relationship between EF and mind-reading, as assessed in the False Belief task, has drawn many researchers' attention. In particular, DCCS performance, or cognitive shift, is significantly related to performance on the Contents False Belief task (Frye et al., 1995), even after controlling for individual differences in verbal ability (Carlson and Moses, 2001). It has been suggested that EF plays a central role in Theory of Mind development. In the False Belief task, the ability to perform a cognitive shift might be necessary to understand others' mental states based on a third-party situation. A related question is whether children better able to perform a cognitive shift would more effectively disambiguate the informative intention of a conversational partner.

The aims of the present study were to investigate the relationship between the ability to follow an explicit topic shift and the ability to perform a cognitive shift as measured by the DCCS. In addition, to appropriately assign the ambiguous referent, the receiver was required to follow the preceding context in accordance with the partner. We specifically examined whether children who were able to perform the cognitive shift necessary to follow another's attention would assign the appropriate referent to the ambiguous utterance. Therefore, we used reference assignment accuracy to investigate the development of disambiguation and cognitive shift ability.

## Materials and methods

### Participants

A total of 44 children (3-years-old: 11 girls, 9 boys; *M* = 42.5 months, *SD* = 3.20 months, and 5-years-old: 13 girls, 11 boys; *M* = 66.2 months, *SD* = 3.71 months) participated in this experiment. None of the children had participated in our previous study (Murakami and Hashiya, in preparation). All of the participants were born full-term and were healthy at the time of the study. Informed consent was obtained from the parents of all the children who participated. An additional four children who were 3 years of age were tested, but excluded from the final sample for the following reasons: understanding of color names was not confirmed (1), obvious bias when answering the questions (100% shape: 1, and 100% color: 1), and noncompliance with the reference assignment task (1).

### Materials and design

Participants were tested individually in a room in the daycare center or preschool they attended. After establishing a rapport with the experimenter, the child participated in a test session. In a test session, the reference assignment task was always presented first. The entire experimental session lasted about 15 min, and all sessions were video recorded.

#### Reference assignment task

***Stimuli***. Laminated cards (14.8 × 21 cm) were used as stimuli. Each card represented one of five kinds of illustrations (umbrella, shoe, chair, cup, or car) painted in one of four colors (red, blue, yellow, or green). One stimulus set included all possible combinations of the objects and colors for a total of 20 cards (five shapes × four colors).

***Procedure***. One test session of the reference assignment task consisted of four trials. A trial consisted of five events, each of which included an explicit question (EQ) or an implicit question (IQ). In an EQ, participants were asked about either the shape or the color of the illustration on the card [“What's (the name of) this?” or “What color is this?”]. In an IQ, participants were asked, “How about this?” The sequence of events included in a trial was as follows: the first event was always an EQ followed by an IQ (PreS-IQ). Another EQ (ESQ) was then asked, but the dimension (shape/color) differed. The ESQ was then followed by two IQs (PostS-IQ1, 2). Half of the four trials began with an EQ about the shape, whereas the other half of the trials began with an EQ about the color. The order of the trials was counterbalanced across participants.

**Figure 1:**
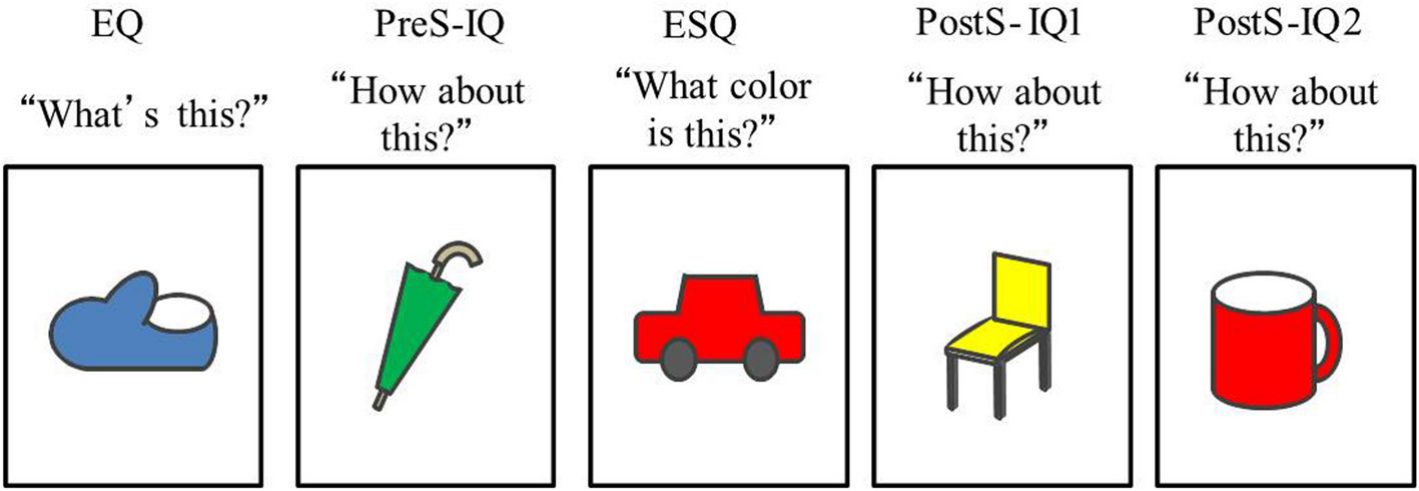
**Schematic sequence of an event in the Reference Assignment task, which includes 5 events in a fixed order of EQ/PreS-IQ/ESQ/PostS-IQ1/PostS-IQ2**.

The child was shown a card, and the experimenter said, “Now, let's try a game. Listen to me carefully and answer the questions.” The experimenter continued to ask questions one at a time about the five cards (see Figure 1). The experimenter made eye contact with the children, and nodded regardless of whether the child had correctly answered the question(s). After asking questions about the five cards, the experimenter aligned the cards in front of the child to indicate to the child that one trial had been completed. The experimenter then took out a new set of cards and began the next trial. A total of four trials were conducted with each child.

***Scoring***. Responses for each trial were coded on a dichotomous rating, defined as follows. For EQs, an appropriate answer was coded as 1, and an incorrect answer was coded as 0 (e.g., an answer that referred to the “color” aspect when the child was asked about an object's “shape” was scored as 0). For IQs, the retrospective answer that referred to the dimension of the explicit question asked just before the implicit question was coded as 1. In addition, a coding battery was applied to the analysis in order to describe the sequential pattern of the child's response beyond a single event.

***Base-Assignment Score***. When both the EQ and PreS-IQ were coded as 1, the Base-Assignment score was coded as 1, reflecting that the child had “appropriately” identified the reference in the absence of a topic shift.

***Shift Score***. The Shift score indicates a child's ability to switch to the explicit question; therefore, this score was coded as 1 when both the EQ and ESQ were coded as 1.

***Re-Assignment Score***. The Re-Assignment score denotes a child's referential assignment based on topic shift; therefore, the score was coded as 1 when both the ESQ and Post-IQ1 were coded as 1.

***Follow-Re-Assignment (Follow-RA) Score***. The Follow-RA score indicates whether the child interpreted the repetition of the same ambiguous question consistently; therefore, the score was coded as 1 when both the PostS-IQ1 and PostS-IQ2 were coded as 1.

#### Dimensional change card sort task

The procedure of the DCCS was consistent with that of Kirkham et al. (2003).

***Stimuli***. The model cards consisted of two white laminated cards (10.5 × 7.5 cm); one card depicted a red truck and the other depicted a blue star. The sorting cards were the same size and shape as the model cards, but each depicted a blue truck or red star. Thus, no sorting card matched a model card in both color and shape. A sorting card was mounted over the bin of each box. The children were trained to sort by color with training cards that depicted blue or red caps, and were trained to sort by shape with training cards that depicted yellow cars or stars.

***Procedure***. The child was shown the two sorting boxes with the model cards. The experimenter then introduced the child to the training part of the game, which consisted of sorting cards that were similar in only one dimension (i.e., cards depicting blue and red caps for the color game or cards depicting yellow cars and stars for the shape game).

The first dimension on which children were trained was counterbalanced across children within each age × gender. Each child was given between 4 and 8 cards (i.e., allowing for four errors). Two cards of one dimension were presented first, followed by two cards of another dimension. Children had to correctly sort four cards (two for each dimension) to pass the training phase. Feedback was provided to the children. The last dimension sorted during the training phase was always the first dimension administered during the test trials (e.g., if the final training card sorted depicted red caps, then the first test dimension would be color). The test trials started immediately after the child had completed the training trials.

There was a minimum of 12 test trials (i.e., six consecutive trials for the first dimension, and six consecutive trials for the second dimension). Because children were required to sort six trials in a row to reach criterion, additional trials were administered until the child passed criterion for that dimension. Additional trials were needed on only two occasions: two 3-years-old children required 6 (1) and 7 trials (1), and one 5-years-old child required 8 trials to reach criterion on the first dimension. The same pseudo-random order of card presentation was used for all children. Before each trial, the child was asked to tell the experimenter the rules of the current game by pointing to the appropriate boxes in response to “knowledge” questions (e.g., “Where do the red ones go in the color game? Where do the blue ones go?”). During alternating trials, the experimenter typically stated the rules and had the child answer the knowledge questions. We randomly varied the value (e.g., red or blue) that was mentioned first.

Children were given feedback on their response to the knowledge question. If the child's response was correct, the experimenter said, “Excellent!” or “Very good.” The child was then given the next card and asked to sort it according to the appropriate dimension (e.g., “Here's a blue one. Where does it go?” or “Here's a car. Where does it go?”). If the child answered the knowledge question incorrectly, the experimenter restated the rules and asked the knowledge question again. If the child responded incorrectly again, the error was noted and the next trial commenced.

Note that the experimenter indicated only the relevant dimension of each stimulus (“Here's a blue one”), whereas in their early work, Zelazo et al. (1996) labeled both dimensions of each stimulus (“Here is a blue car”). In addition, feedback was not provided to the child during testing. The child was asked to place the sorting cards face down in the sorting boxes. After the child had correctly sorted six cards by the first dimension, the sorting dimension was switched. Moreover, children were allowed to self-correct.

Then, based on their DCCS performance, children were divided into two groups: DCCS-passed and DCCS-failed. To pass the DCCS, children must correctly sort five of the six cards. We examined whether the children who passed the DCCS showed better performance on the reference assignment task than the children who failed the DCCS; therefore, we used this classification as a categorical factor on the reference assignment task.

## Results

### Reference assignment task

For the reference assignment task, preliminary analysis revealed no gender differences or effect of trial order; thus, these factors were collapsed in the subsequent analyses. Table 1 shows the mean score for each event in the reference assignment task. The averaged score for each event was compared in a 2 × 2 ANOVA with Age (3 vs. 5 years) as a between-subjects factor and Event (Base-Assignment vs. Shift vs. Re-Assignment vs. Follow-RA) as a within-subjects factor. The results revealed a significant interaction between Age and Event [*F*_(3, 126)_ = 3.71, *p* = 0.01, η^2^_*p*_ = 0.08] and a significant main effect of Age and Event [Age: *F*_(1, 42)_ = 22.93, *p* < 0.001, η^2^_*p*_ =0.35; Event: *F*_(3, 126)_ = 18.07, *p* < 0.001, η^2^_*p*_ = 0.30]. Multiple comparisons revealed that 5-year-old outperformed 3-years-old except in the Base-Assignment score (*p* < 0.01 at maximum). Moreover, in 3-years-old, Base-Assignment (*M* = 3.2, *SD* = 0.88) included more “appropriate” answers than other scores on Shift (*M* = 2.4, *SD* = 1.19), Re-Assignment (*M* = 2.0, *SD* = 0.65) and Follow-RA (*M* = 2.0, *SD* = 0.83), *p* < 0.01 at maximum. In 5-years-old, Base-Assignment (*M* = 3.4, *SD* = 0.78) and Shift (*M* = 3.7, *SD* = 0.55) included more “appropriate” answers than Re-Assignment (*M* = 2.8, *SD* = 0.87) and Follow-RA (*M* = 2.7, *SD* = 0.86), *p* < 0.01 at maximum. The age-dependent patterns observed in the present study are consistent with those of our previous study (Murakami and Hashiya, in preparation).

**Table 1:** **Mean score and standard deviation (in parentheses) for each event of the Referential Assignment task**.

	**Base-assignment**	**Shift**	**Re-assignment**	**Follow-RA**
3-years-old	3.2 (0.88)	2.4 (1.19)	2.0 (0.65)	2.0 (0.83)
5-years-old	3.4 (0.78)	3.7 (0.55)	2.8 (0.87)	2.7 (0.86)

**Figure 2:**
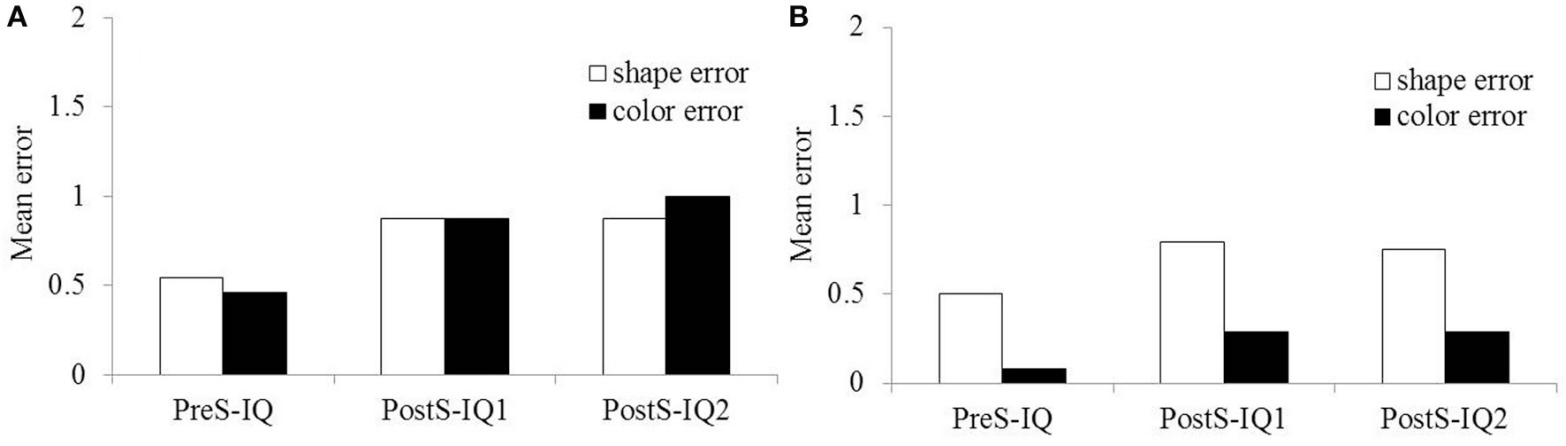
**Mean error for Implicit Questions in (A) 3-years-old and (B) 5-years-old**.

To examine whether there was a bias to respond to a specific aspect when presented with ambiguous questions, we tallied the number of errors for shifts from color to shape (0–2), or shape to color, during each event for the two age groups. Figure 2 shows the mean error for IQs in 3- and 5-years-old. The results of a *t*-test for each event suggested there was no difference in 3-years-old children [PreS-IQ, *t*_(17)_ = 1.000, *p* = 0.33, *r* = 0.24; PostS-IQ1, *t*_(17)_ = 0.579, *p* = 0.57, *r* = 0.14; PostS-IQ2, *t*_(17)_ = 0.437, *p* = 0.66, *r* = 0.11]; however, 5-years-old children were more likely to answer in the shape than the color when an ambiguous question was presented [PreS-IQ, *t*_(23)_ = 2.632, *p* = 0.01, *r* = 0.48; PostS-IQ1, *t*_(23)_ = 2.077, *p* = 0.049, *r* = 0.40; PostS-IQ2, *t*_(23)_ = 1.967, *p* = 0.06, *r* = 0.38]. The response bias observed in 5-years-old is inconsistent with our previous research (Murakami and Hashiya, in preparation). Although 5-years-old tended to state the shape of the object in response to an ambiguous question, the exact error rate (29–40%) remained within the range of chance; thus, the results may not have necessarily indicated a shape bias (Landau et al., 1988). Therefore, we did not consider this a significant reaction characteristic of 5-years-old and continued the analysis.

### Dimensional change card sort task

For the DCCS, all children sorted all the cards correctly for the first dimension. After the switch to the second dimension, 93% of the children consistently sorted all the cards correctly, or all incorrectly. Given the lack of variance, nonparametric categorical analyses (chi-square) were used to analyze the data. The number of children who successfully switched dimensions in the card-sorting task is shown in Table 2. The majority of 3-years-old performed poorly (only 25% successfully switched dimensions), while most 5-years-old performed well (66% successfully switched dimensions). The difference in performance between the two groups was significant [χ^2^_(*df* = 1, *N* = 44)_ = 6.013, *p* < 0.05]. These results suggest that the current sample was similar to those of previous studies.

**Table 2:** **Distribution of the group of age × performance on DCCS**.

**Age**	**DCCS**	***N* (girls)**	**Mean months**	***SD***
3	Failed	15 (8)	42.2	3.3
	Passed	5 (3)	43.2	2.9
5	Failed	8 (6)	64.9	4.4
	Passed	16 (7)	66.9	3.3

### Comparison between the reference assignment task and DCCS task

**Figure 3:**
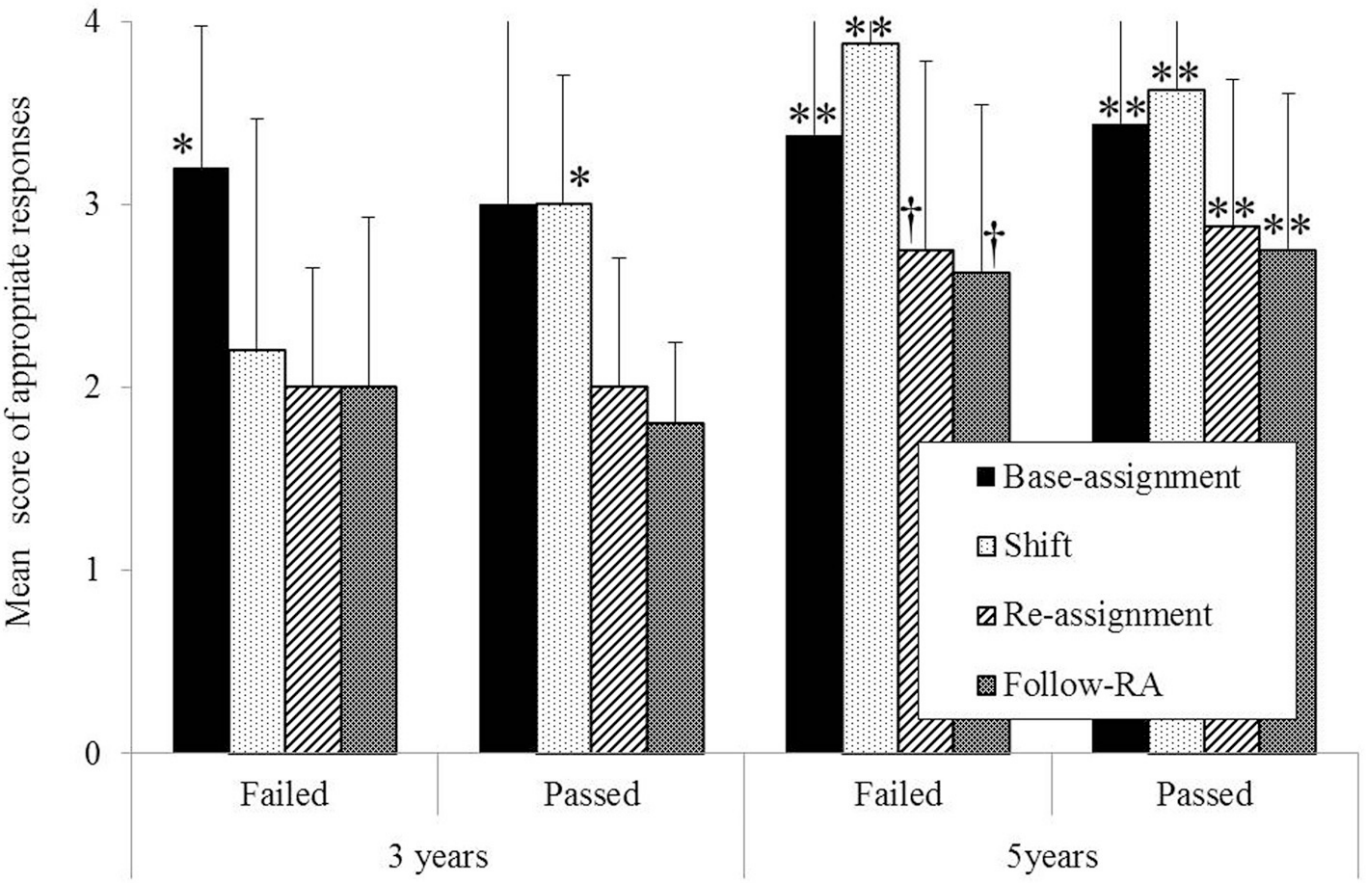
**Mean score of appropriate responses**. ^**^,^*^ and ^†^indicate that the score was above chance level (=2), *p* < 0.01, 0.05, and 0.10, respectively.

The number of “appropriate” responses in the reference assignment task was analyzed using a 2 × 2 × 4 mixed ANOVA with Age (3 vs. 5 years) and DCCS group (passed vs. failed) as between-subjects factors, and Event (Base-Assignment vs. Shift vs. Re-Assignment vs. Follow-RA) as a within-subjects factor. No significant interactions between factors were found (see Figure 3); however, main effects of Age and Event [Age: *F*_(1, 40)_ = 16.48, *p* < 0.001, η^2^_*p*_ = 0.28; Event: *F*_(3, 120)_ = 16.59, *p* < 0.001, η^2^_*p*_ = 0.34] were observed. The main effect of DCCS was not significant.

To determine the rate of correct responses to the questions, the proportion of appropriate responses was compared with chance levels (=2). For the 3y-failed group, one-sample *t*-tests indicated that performance was above chance level for the Base-Assignment score [*t*_(14)_ = 6.00, *p* < 0.001, *r* = 0.85], but performance in other events remained within the range of chance. One-sample *t*-tests for the 3y-passed group indicated that performance was above chance level only for the Shift questions [*t*_(4)_ = 3.16, *p* = 0.034, *r* = 0.85]. On the other hand, analysis of 5y-failed group indicated that performance was above chance level for all events [Base-Assignment; *t*_(7)_ = 4.25, *p* = 0.004, *r* = 0.85; Shift; *t*_(7)_ = 15.00, *p* < 0.0001, *r* = 0.99; Re-Assignment; *t*_(7)_ = 2.05, *p* = 0.08, *r* = 0.61; Follow-RA; *t*_(7)_ = 2.05, *p* = 0.095, *r* = 0.61]. Analysis of the 5y-passed group also indicated that performance was above chance level for all events [Base-Assignment; *t*_(15)_ = 7.90, *p* < 0.001, *r* = 0.90; Shift; *t*_(15)_ = 10.50, *p* < 0.001, *r* = 0.94; Re-Assignment; *t*_(15)_ = 4.34, *p* = 0.001, *r* = 0.75; Follow-RA; *t*_(15)_ = 3.50, *p* = 0.003, *r* = 0.67].

## Discussion

The current study directly compared performance on a reference assignment task with DCCS performance in preschoolers, and identified a relationship between the ability to follow an explicit utterance and the ability to perform a cognitive shift, which develops between 3 and 5 years of age (Zelazo et al., 1996, 2003; Carlson and Moses, 2001; Kirkham et al., 2003; Müller et al., 2006; Moriguchi et al., 2007; Moriguchi and Hiraki, 2009). However, the present findings indicate that some aspects of the ability to disambiguate based on prior verbal exchanges do not always reflect a cognitive shift. A previous study showed that children interpret the ambiguous speech of others by referring to information from a prior situation in which one potential referent was salient (Murakami and Hashiya, in preparation). In the reference assignment task, children in the current study replicated this finding. Performance on the DCCS was also consistent with the previously observed patterns for these age groups. These results suggest that the participant group in the current study did not differ qualitatively from those of previous studies.

The comparison of these two tasks contributes to our knowledge of the relationship between EF and understanding verbal instruction. On the Shift score, although the ANOVA results did not show an Age × DCCS interaction, a comparison with chance level showed that the 3-years-old children who passed the DCCS effectively redirected their attention in response to explicit verbal instruction. These results suggest that the ability to focus on another aspect of a target in response to language is necessary to shift the classification rule, such as in the DCCS. However, even though they could shift their explicit attention, the 3-years-old children who passed the DCCS did not retrospectively assign the referent based on the preceding explicit verbal exchange. These results suggest that the cognitive ability of shifting attention does not always facilitate the retrospective reference.

In a similar fashion, both groups of 5-years-old children showed only moderate performance in ESQ, even though it was above chance level. However, their verbal shifting performance seemed to show a ceiling effect. This inconsistency suggests that the difficulties in nonverbal shifting are not tightly related to verbal shifting ability, which might be consistent with previous findings about the knowledge questions of the DCCS (Kirkham et al., 2003), which are structurally similar to the ESQ in the reference assignment task. In addition, the ceiling effect in 5-years-old might be explained by a developmental improvement in sensitivity toward verbal instruction.

Moreover, the DCCS-failed group of 5-years-old children also showed a marginal tendency to retrospectively reference. When we compared Re-Assignment and Follow-RA scores with chance level, we found that both groups of 5-years-old children disambiguated the ambiguous deictic; they tended to interpret the ambiguous utterance retrospectively. These results suggest that even the children who failed the DCCS could disambiguate the ambiguous utterance.

The reference assignment task, which enables systemic assessment of one's understanding of a deictic, potentially represents a means of separating underlying systems that mediate the process of disambiguation. Further, the current results demonstrate that the ability of cognitive shift is correlated with the ability to disambiguate the linguistic referent, but only to a limited extent.

Thus, the results did not support the expectation that the ability of cognitive shift would entirely explain the ability to disambiguate a linguistic referent, but rather suggested independent development of retrospective referencing and cognitive shift.

The ability to use contextual information from a shared conversational background is one of the essential pragmatic skills (Clark and Marshall, 1981) in effectively inferring the references of another (Sperber and Wilson, 2002). Though previous findings have demonstrated that even 2-years-old infants interpret an ambiguous request for an object in terms of prior interactions with the requestor (Ganea and Saylor, 2007; Saylor and Ganea, 2007), the current study suggested the difficulty for 3-years-old children in identifying an ambiguous referent based only on verbal information. Considering these concerns, our results may imply that several extra processes are required for completing our reference assignment task: the processes such as acquisition of a semantic definition of the deictic, or the conventional principle that the ambiguous “this” embedded in a specific form of the sentence refers to some salient aspect or event expressed in the precedent utterance, should be the candidates for such missing pieces. Based on the current findings, the detailed interactions of such contributing factors should be a focus of future studies. Thus, studies geared toward dissecting the development of pragmatic communication might serve as an effective means of describing the generality and specificity of the development of EF, especially when the reference assignment task is included in the test battery.

### Conflict of interest statement

The authors declare that the research was conducted in the absence of any commercial or financial relationships that could be construed as a potential conflict of interest.
